# Explaining clinical behaviors using multiple theoretical models

**DOI:** 10.1186/1748-5908-7-99

**Published:** 2012-10-17

**Authors:** Martin P Eccles, Jeremy M Grimshaw, Graeme MacLennan, Debbie Bonetti, Liz Glidewell, Nigel B Pitts, Nick Steen, Ruth Thomas, Anne Walker, Marie Johnston

**Affiliations:** 1 Institute of Health and Society, Newcastle University, Baddiley-Clark Building, Richardson Road, Newcastle upon Tyne, NE2 4AX, United Kingdom; 2 Clinical Epidemiology Programme, Ottawa Health Research Institute and Department of Medicine, University of Ottawa, 1053 Carling Avenue, Administration Building Room 2-017, Ottawa, ON, K1Y 4E9, Canada; 3 Health Services Research Unit, University of Aberdeen, Foresterhill, Aberdeen, AB25 2ZD, United Kingdom; 4 Dental Health Services & Research Unit, University of Dundee, MacKenzie Building, Kirsty Semple Way, Dundee, DD2 4BF, United Kingdom; 5 Leeds Institute of Health Sciences, Charles Thackeray Building, University of Leeds, 101 Clarendon Road, Leeds, LS2 9LJ, United Kingdom; 6 College of Life Sciences and Medicine, University of Aberdeen, Health Sciences Building (2nd floor), Foresterhill, Aberdeen, AB25 2ZD, United Kingdom

## Abstract

**Background:**

In the field of implementation research, there is an increased interest in use of theory when designing implementation research studies involving behavior change. In 2003, we initiated a series of five studies to establish a scientific rationale for interventions to translate research findings into clinical practice by exploring the performance of a number of different, commonly used, overlapping behavioral theories and models. We reflect on the strengths and weaknesses of the methods, the performance of the theories, and consider where these methods sit alongside the range of methods for studying healthcare professional behavior change.

**Methods:**

These were five studies of the theory-based cognitions and clinical behaviors (taking dental radiographs, performing dental restorations, placing fissure sealants, managing upper respiratory tract infections without prescribing antibiotics, managing low back pain without ordering lumbar spine x-rays) of random samples of primary care dentists and physicians. Measures were derived for the explanatory theoretical constructs in the Theory of Planned Behavior (TPB), Social Cognitive Theory (SCT), and Illness Representations specified by the Common Sense Self Regulation Model (CSSRM). We constructed self-report measures of two constructs from Learning Theory (LT), a measure of Implementation Intentions (II), and the Precaution Adoption Process. We collected data on theory-based cognitions (explanatory measures) and two interim outcome measures (stated behavioral intention and simulated behavior) by postal questionnaire survey during the 12-month period to which objective measures of behavior (collected from routine administrative sources) were related. Planned analyses explored the predictive value of theories in explaining variance in intention, behavioral simulation and behavior.

**Results:**

Response rates across the five surveys ranged from 21% to 48%; we achieved the target sample size for three of the five surveys. For the predictor variables, the mean construct scores were above the mid-point on the scale with median values across the five behaviors generally being above four out of seven and the range being from 1.53 to 6.01. Across all of the theories, the highest proportion of the variance explained was always for intention and the lowest was for behavior. The Knowledge-Attitudes-Behavior Model performed poorly across all behaviors and dependent variables; CSSRM also performed poorly. For TPB, SCT, II, and LT across the five behaviors, we predicted median R^2^ of 25% to 42.6% for intention, 6.2% to 16% for behavioral simulation, and 2.4% to 6.3% for behavior.

**Conclusions:**

We operationalized multiple theories measuring across five behaviors. Continuing challenges that emerge from our work are: better specification of behaviors, better operationalization of theories; how best to appropriately extend the range of theories; further assessment of the value of theories in different settings and groups; exploring the implications of these methods for the management of chronic diseases; and moving to experimental designs to allow an understanding of behavior change.

## Background

In the field of implementation research, there are problems with trying to understand the effect of interventions to change healthcare professional behavior. When the effects of interventions are retrospectively examined within systematic reviews [1,2], it is difficult to understand or explain the variation in effect sizes that are often seen. Given the increasing interest in understanding the causal mechanisms of action within interventions [3,4], there is an increased interest in the use of theories of behavior when designing implementation research studies to change behavior. Historically, theory has not been widely used; when analysing a systematic review of guideline implementation studies Davies et al. concluded that ‘there was poor justification of choice of intervention and use of theory in implementation research in the identified studies until at least 1998’ [5]. Although there are some terminological debates (reflecting the several ways in which theory can be used—being theory-based as opposed to theory-inspired), using theory has a number of clear advantages: it allows a view of why/how behaviors change; it offers an approach to how interventions can be developed; it can guide analysis; and it offers a framework to support generalizability [3,6].

Having articulated a role for theory, a researcher then faces the next decision of ‘which theory’? To date, groupings of theories have been examined within systematic reviews (e.g., social cognitive theories [7]), they have been aggregated into overarching theories (almost meta-theories) [8], or, at least in part to deal with the plethora of available options, they have been grouped and then reconfigured into common underlying conceptual domains [9]. Against this evolving backdrop, in 2003 we initiated a series of five studies to establish a scientific rationale for interventions to translate research findings into clinical practice. We explored the performance of a number of different, commonly used, and overlapping theories and models [10-14]. Working across five behaviors and two disciplines of healthcare professionals, the studies were novel, and demanding to conduct, analyse, and interpret. Having completed the analyses of the individual studies, in this paper, we present an overview of the methods and results across the five conditions and multiple theories. We reflect on the performance of the theories, the strengths and weaknesses of the method, and consider where these methods sit alongside the range of methods for studying healthcare professional behavior change.

## Methods

The methods of the studies (along with the questionnaires used) are described in detail elsewhere [10-14]. Four of the analyses have been previously reported [11-14]; we report the fifth analysis in Additional file 1 and include the questionnaire used as Additional file 2.

In summary, these were five predictive studies exploring theory-based cognitions as predictors across five different clinical behaviors within a series of random samples of primary care dentists and primary care physicians in Grampian, Tayside and Lothian in Scotland and Durham, Newcastle and South Tees in northern England. Data on theory-based cognitions (explanatory measures) and two interim outcome measures (stated behavioral intention and simulated behavior) were collected by a single postal questionnaire survey that took place during the 12-month period to which data on objective measures of behavior (collected from routine administrative sources) related.

Planned analyses explored the predictive value of theories and theory-based cognitions in explaining variance in the behavioral data.

### Explanatory measures and theories

Explanatory measures were chosen to reflect both volitional (processes that are aware, deliberative, reflective, and may therefore be considered subject to volition) and non-volitional (processes that are associative, automatic, impulsive, and may therefore be considered not subject to volition; here represented by Learning Theory, or LT) theoretical constructs that have been found to predict behavior. All measures were included in each study. Measures were derived for the constructs in the Theory of Planned Behavior (TPB) [15], Social Cognitive Theory (SCT) [16,17], and for illness representations specified by the Common Sense Self Regulation Model (CSSRM) [18]. We constructed self-report measures of two constructs from LT (habitual behavior and anticipated consequences) [19,20], a measure of implementation intentions (II) [21], and questions to assess stage in the process of change as specified by the Precaution Adoption Process (PAP) [22,23].

The TPB [15] by Aizen and Fishbein proposes that the strength of an individual’s behavioral intention to engage in a behavior, and the degree of control they feel they have over that behavior (perceived behavioral control) are the proximal determinants of engaging in it. The perceived behavioral control construct is closely related to the concept of self-efficacy in SCT. The theory of planned behavior also proposes that behavioral intention strength is determined by three variables: attitudes towards the behavior (the extent to which the behavior will result in consequences that the person values); subjective norms for the behavior (the belief that other people whose opinion influences the person think the person should engage in the behavior); and perceived behavioral control over it. These variables in turn are based upon salient behavioral, normative, and control beliefs about the behavior. Each predictor variable may be measured directly (by asking respondents about their overall attitude), or indirectly (by asking respondents about specific behavioral beliefs and outcome evaluations). Aizen suggests that behavior change is brought about by interventions that change these beliefs.

Bandura’s SCT [16] proposes that behavior is determined by three kinds of expectancies: situation-outcome expectancies, outcome expectancies, and self-efficacy expectancies. Situation-outcome expectancies are beliefs about how events are connected (e.g., a diagnosis of cancer is likely to result in death). Outcome expectancies refer to beliefs about the consequences of performing a behavior (e.g., if I stop smoking, I will put on weight). Self-efficacy expectancies are beliefs about one’s ability to perform the behavior (e.g., I can stop smoking). Self-efficacy has been found to be the strongest determinant of behavior, and the theory proposes four methods of changing self-efficacy in order to change behavior: providing mastery experiences (i.e., successful behavioral performance); modelling (i.e., observation of successful behavioral performance); verbal persuasion; and addressing attributions for physiological and affective states

Leventhal’s CSSRM [18] proposes that individuals attempt to make sense of illness by making use of pre-existing knowledge or schemas to develop both cognitive representations of the illness (including its identity, consequences, timeline, cause, and cure/control) and emotional representations of the threat involved. These representations give rise to behavioral responses. For patients, behavioral responses may include going to see a doctor, taking prescribed or non-prescribed medicines, or participating in rehabilitation programmes. For clinicians, responses may include referring a patient for diagnostic tests, prescribing a drug, or restoring a carious tooth. The model is described as self-regulatory because this is seen as a dynamic process. The aim of the actions that people take in response to a health threat is to reduce the degree of threat or to restore their own (or their patient’s) emotional equilibrium. The individual’s own understanding of the health condition and their personal emotional reaction are central to the self-regulatory approach. It seems likely that health professionals also experience emotional reactions to some conditions (e.g., terminal conditions in children) or patient groups, for example ‘heart sink’ reactions, which may influence their practice. In this model, behavior change results from changes in the cognitive representations or in the responses available to manage either the threat or the emotions.

Learning theory proposes that behaviors that have positive consequences for the individual (such as remuneration) are likely to be repeated, whereas those that have unpleasant consequences will become less frequent [20]. Learning happens when consequences occur if and only if the behavior has occurred and can take a variety of forms, from material rewards (e.g., financial incentives), through social rewards (e.g., receiving praise) to personally salient rewards (e.g., achieving a desired goal). The principle that positive consequences promote repetition of behavior is well established and has been widely and successfully used to understand behavior and behavior change [24]. As behaviors are repeatedly rewarded, they may become ‘habitual’ and, if reward is scheduled appropriately, the behavior may be maintained with a reduced frequency of reward; if a behavior is increased by being rewarded and then removed without planned scheduling, the learned behavior will be ‘extinguished’ unless some other form of reward is available,

‘Implementation Intentions’ are explicit ‘if-then’ action plans about when and where a goal intention will be achieved [21]. Gollwitzer [21] has made the distinction between goal intentions and II. A goal intention is an intention to perform a behavior or achieve a goal (e.g., I intend to reduce the number of referrals I make for lumbar spine x-rays). This is conceptually close to the behavioral intention construct in the TPB. Gollwitzer argues that by creating an II, people effectively transfer control of the behavior to the environment—establishing cues to action. For example, by saying that ‘When a patient tells me about their low back pain, I will explain the pros and cons of an x-ray to them.’

Stage theories propose that behavior change occurs in a stepwise process, rather than a linear fashion as implied by motivational or action theories. From a stage theory perspective, interventions to facilitate change will be most effective if they are tailored to the stage an individual has reached within this process. While there are differences between the stage models in the number and nature of stages proposed, stage theories typically distinguish motivation and action steps [17,22,25]. In the PAP model, Weinstein has proposed an additional early stage when individuals may be unaware of the need for behavior change, a stage which may have particular relevance to the early stages of the implementation of evidence [22].

A common implicit model is the Knowledge-Attitudes-Behavior model. This assumes that a change in knowledge will produce a change in attitude, and this will, in turn, produce a change in behavior. While this model has some value, it should be tested rather than assumed before being used as a basis for behavior change. Accordingly, we examined the ability of knowledge to predict behavior.

The questions were developed based on the standard methods for each theory where possible. Additionally, for each behavior five knowledge questions, for which there was good evidence, were developed by the study team. Table 1 provides a summary of the explanatory measures used in each of the studies (with examples from the Managing Low Back Pain Without Ordering Lumbar Spine X-rays study). Questions were rated on a seven-point Likert scale from ‘strongly disagree’ to ‘strongly agree.’ Scores were adjusted so that a higher score equated with a greater propensity towards evidence-based behavior. Exemplar questionnaires are available through previous publications [11,13,14] and in Additional file 2.

**Table 1:** Summary of the explanatory measures

**Constructs (number of questions)**	**Example Question(s)**
**Theory of Planned Behavior**[15]	
Behavioral intention (3)	I intend to refer patients with back pain for an X-ray as part of their management
Attitude: Direct (3); Indirect^a^ (8 behavioral beliefs (bb) multiplied by 8 outcome evaluations (oe). The score was the mean of the summed multiplicatives.)	Direct: In general: The possible harm to the patient of a lumbar spine X-ray is outweighed by its benefits; Indirect: In general, referring patients with back pain for an X-ray would reassure them (bb) x reassuring patients with back pain is (oe: un/important)
Subjective Norm: Indirect (4 normative beliefs (nb) multiplied by 4 motivation to comply (mtc) questions. The score was the mean of the summed multiplicatives).	I feel under pressure from the NHS not to refer patients for an X-ray (nb) x How motivated are you to do what the NHS thinks you should (mtc: very much/not at all)
Perceived Behavioral Control: Direct (4); Indirect/power (14)^c^	Direct: Whether I refer patients for a lumbar X-ray is entirely up to me. Indirect: Without an X-ray, how confident are you in your ability Not at all Extremely to treat patients with back pain who: Expect me to refer them for an X-ray
**Social Cognitive Theory**[16]	
Risk Perception (3)	It is highly likely that patients with back pain will be worse off if I do not refer them for an X-ray.
Outcome Expectancies Self (2 × 2), Behavior (8x8). The score was the mean of the summed multiplicatives.	Self: If I refer a patient with back pain for an X-ray, then I will think of myself as a competent GP x Thinking of myself as a competent GP is (Un/Important) Behavior: See Attitude (Theory of Planned Behavior)
Self Efficacy: General: Generalized Self-Efficacy Scale [17] (10: 4 point scale, not at all true/exactly true); Specific (7)	General: I can always manage to solve difficult problems if I try hard enough Specific: How confident are you in your ability to treat back problems without using an X-ray report
**Implementation Intention**[21]	
Action planning (3)	Currently, my standard method of managing patients with back pain does not include referring them for an X-ray
**Learning Theory**[19,20]	
Anticipated consequences (3)	If I start routinely referring patients with back pain then, on balance, my life as a GP will be easier in the long run
Evidence of habit (2)	When I see a patient with back pain, I automatically consider referring them for an X-ray
Experienced (rewarding and punishing) consequences (4: more likely to refer (score = 1); less likely (score = -1); unchanged/not sure/never occurred (score = 0)). Scores were summed.	Think about the last time you referred a patient for a lumbar spine X-ray and felt pleased that you had done so. Do you think the result of this episode has made you: Think about the last time you decided not to refer a patient for a lumbar spine X-ray and felt sorry that you had not done so. Do you think the result of this episode has made you:
**Common Sense Self Regulation Model**^**d**^[18]	
Perceived identity (3)	Back pain as seen in general practice is generally of an intense nature
Perceived cause (8)	Back pain is caused by stress or worry
Perceived controllability (7)	What the patient does can determine whether back pain gets better or worse, What I do can determine whether the patient’s back pain gets better or worse
Perceived duration (5)	Back pain as seen in general practice is very unpredictable
Perceived consequences (3)	Back pain does not have much effect on a patient’s life
Coherence (2)	I have a clear picture or understanding of back pain
Emotional response (4)	Seeing patients with back pain does not worry me
**Precaution Adoption Process (Stage model) **[22]; [21]	
Current stage of change. A single statement is ticked to indicate the behavioral stage	Unmotivated (3): I have not yet thought about changing the number of lumbar X-rays I currently request. It has been a while since I have thought about changing the number of lumbar X-rays I request. Motivated (2): I have thought about it and decided that I will not change the number of lumbar X-rays I request. I have decided that I will request more lumbar X-rays. I have decided that I will request less lumbar X-rays. Action (1): I have already done something about increasing the number of lumbar X-rays I request I have already done something about decreasing the number of lumbar X-rays I request
**Other Measures**	
Knowledge (5) (True/False/Not Sure)	The presence of spondolytic changes on a lumbar spine X-ray correlates well with back pain
Demographic	Post code, gender, time qualified, number of other doctors in practice, trainer status, hours per week, list size

^a^ All indirect measures consist of specific belief questions identified in the preliminary study as salient to the management of low back pain.

^b^ These individuals and groups were identified in the preliminary study as influential in the management of low back pain.

^c^ An indirect measure of perceived behavioral control usually would be the sum of a set of multiplicatives (control beliefs x power of each belief to inhibit/enhance behavior). However, the preliminary study demonstrated that it proved problematic to ask clinicians meaningful questions which used the word ‘control’ as clinicians tended to describe themselves as having complete control over the final decision to perform the behavior. Support for measuring perceived behavioral control using only questions as to the ease or difficulty of performing the outcome behavior was derived from a meta-nalysis which suggested that perceived ease/difficulty questions were sensitive predictors of behavioral intention and behavior (Trafimow et al., 2002).

^d^ Illness representation measures were derived from the Revised Illness Perception Questionnaire (Moss-Morris, R., Weinman, J., Petrie, K. J., Horne, R., Cameron, L.D., & Buick, D. 2002).

### Behaviors, simulated behavior, and behavioral intention (dependent variables)

The three dental and two medical behaviors are shown in Table 2. In each case, the description of the clinical behavior contained in the questionnaires was specified according to the TACT principle—in terms of its Target patient, the Action to be performed, the Context or conditions for the action and when the action would be performed (Time). For example, for managing low back pain, the target is patients presenting with back pain, the action is managing the patient without referring for lumbo-sacral spine (lumbar) X-rays, the context is general practice, and the time is (implicitly) now.

**Table 2:** Description of the five study behaviors, definition and mean (SD) rates of performing the objective measures of these behaviors, mean (SD) simulated behavior scores and mean (SD) intention scores

**Study Behaviors**	**Objective measures of behavior**	**Mean (SD) rates of behavior**	**Simulated behavior Mean (SD) scenario scores (out of 5)**	**Intention Mean (SD) (out of 7)**
**General Dental**				
**Taking Dental Radiographs**: Taking small or medium intra-oral radiographs (not for orthodontic reasons)	Number of intra oral radiographs taken per course of treatment Data obtained from a national fee claims database used for paying dental practitioners	20.3 (9.0) radiographs per 100 courses of treatment	Would take 2.4 (1.2) radiographs	4.8 (1.3)
**Performing Dental Restorations**: The use of restorations in managing caries in permanent teeth in children (patients under 17 years of age)	Number of restorations per 100 courses of treatment Data obtained from a national fee claims database used for paying dental practitioners.	10.4 (4.3) restorations per 100 courses of treatment	Would restore in 2.9 (1.1) cases	4.9 (1.1)
**Placing Fissure Sealants**: Placing a fissure sealant in a 6 to 16 year old patient	Unable to reliably measure behavior given the target age group		Would place fissure sealants for 2.03 (1.54) cases	4.9 (1.2)
**General Medical**				
**Managing URTIs Without Prescribing Antibiotics**: Managing patients presenting with an upper respiratory tract infection (include sore throats, nasal discharge and coughs) in primary care *without* prescribing an antibiotic	Mean number of prescriptions for an antibiotic issued per 100 patients registered with each primary care practice per year Data derived from a national database of issued prescriptions	57 (31) prescriptions per 100 patients registered per year	Would prescribe for 1.6 (1.2) cases	5.8 (0.8)
**Managing Low Back Pain Without Ordering Lumbar Spine X-rays**: Managing patients presenting with uncomplicated low back pain in primary care without referring them for a lumbo-sacral spine x-ray	Mean number of lumbar spine x-rays taken per 1000 patients registered with each primary care practice per year Data derived from the reporting systems of the hospitals where the x-rays were performed	5.0 (8.9) x-rays per 1000 patients registered per year	Would refer for lumbar spine x-ray in 1.5 (1.2) cases	5.9 (1.0)

For each of the five behaviors, we collected two measures of behavior, an objective measure (Table 2) collected from routine data systems and gathered for a 12-month period to control for seasonal variations, and a measure of simulated behavior. For simulated behavior, key elements that may influence clinicians’ decisions to perform the behaviors were identified from the literature, opinion of the clinical members of the research team, and the interviews with clinicians. From this, we constructed five clinical scenarios that described patients presenting in relation to the index behaviors. Respondents were asked to decide what they would do, and responses were summed to create a total score out of a possible maximum of five (Table 1).

We also elicited behavioral intention using three questions assessing intention to perform the behavior of interest. With a behavior specific stem the questions were worded ‘I have in mind to,’ ‘I intend to,’ ‘I aim to’ (rated on a seven-point scale from ‘strongly disagree’ to ‘strongly agree’).

### Procedure

Clinicians were sent an invitation pack comprising a letter of invitation, a questionnaire consisting of theory-based cognition measures (explanatory measures), two interim outcome measures (behavioral intention and behavioral simulation), and demographic measures, plus a consent form to allow access to their data (objective behavior measure) from the routine systems, and a reply-paid envelope. Three postal reminders were sent to non-responders at two, four, and six weeks from the first mailing.

### Statistical analysis

Each study included a large number of predictor variables, and a power calculation suggested that, for each of the five studies, a minimum sample of 200 clinicians was required to detect an effect size of 0.40, with alpha of 0.05, 95% power.

The measures of the theoretical constructs were tested for acceptable internal consistency. If the criterion value (0.60) was not reached, items were dropped from the variable measures until the maximum possible Cronbach’s alpha was achieved or, for two items, a correlation greater than 0.25.

We examined the relationship between predictive and dependent variables using correlation, except for the PAP where ANOVA was used. For the two general medical behaviors for the PAP analysis, respondents were dichotomized into two groups (decided to perform desired behavior or have already performed desired behavior versus all other responses), and the relationship between predictive and outcome variables were examined using regression models.

Multiple regression analyses were used to examine the predictive value of each of the theoretical models. For the behavior ‘managing low back pain without referring for a lumbar spine x-ray,’ given the distribution of the behavioral data, we used negative binomial regression (NBR) to model the predictive ability of individual theoretical constructs and complete theories.

### Ethics approval

The study was approved by the UK South East Multi-Centre Research Ethics Committee (MREC/03/01/03).

## Results

### Response rates

Questionnaire response rates were: 48% (214/450) for taking dental radiographs; 29% (130/450) for performing dental restorations; 29% (120/407) for placing fissure sealants; 21% (230/1100) for managing upper respiratory tract infections (URTIs) without prescribing antibiotics; and 27% (299/1100) for managing low back pain without ordering lumbar spine x-rays. We achieved the target sample size for three of the five studies.

### Summary of scores

The rates of performing the objective behaviors and the scores for simulated behavior and intention are shown in Table 2.

Summaries of the mean scores for the explanatory variables are shown in Table 3 and Additional file 3. In general, the mean scores were above the midpoint on the scale, with median values across the five behaviors generally being above four out of seven and the range being from 1.53 to 6.01.

**Table 3:** Descriptive statistics for Theory of Planned Behavior, Social Cognitive Theory, Implementation Intention, Learning Theory, and Knowledge for each of the five behaviors

**Theory**	**Predictive constructs**	**Taking dental radiographs**				**Performing dental restorations**				**Placing fissure sealants**				**Managing URTIs**				**Managing low back pain**				**Min**	**Med**	**Max**
		**N**	**Alpha**	**Mean**	**SD**	**N**	**Alpha**	**Mean**	**SD**	**N**	**Alpha**	**Mean**	**SD**	**N**	**Alpha**	**Mean**	**SD**	**N**	**Alpha**	**Mean**	**SD**	** of Mean**		
	Attitude direct	2	0.40	5.85	1.00	2	0.37	4.70	1.10	2	0.57	5.64	0.99	3	0.54	5.10	0.93	2	0.25	3.40	1.20	3.40	5.10	5.85
**Theory of Planned Behavior**	Attitude indirect	12	0.75	4.30	0.76	6	0.65	3.31	0.81	7	0.76	4.28	0.95	7	0.56	4.97	0.73	4	0.75	5.34	0.99	3.31	4.30	5.34
	Subjective Norm	5	0.83	1.53	0.91	4	0.77	2.19	0.95	3	0.70	2.13	1.03	3	0.68	5.61	0.90	4	0.68	5.86	0.69	1.53	2.19	5.86
	Intention	3	0.73	4.77	1.27	3	0.79	4.90	1.13	3	0.79	4.90	1.24	3	0.68	5.83	0.83	3	0.69	5.90	1.00	4.77	4.90	5.90
	PBC direct	4	0.76	2.48	1.00	4	0.71	3.58	1.08	5	0.61	4.53	0.96	4	0.70	4.25	1.13	4	0.63	4.50	1.10	2.48	4.25	4.53
	PBC indirect/power	12	0.84	4.09	0.87	10	0.73	3.68	0.81	10	0.80	3.98	0.97	7	0.86	4.51	0.94	14	0.91	4.90	1.00	3.68	4.09	4.90
**Social**	Risk perception	2	0.51	4.60	1.30	3	0.51	4.30	1.00	6	0.60	4.84	0.79	3	0.61	5.07	0.93	2	0.46	5.80	1.00	4.30	4.84	5.80
**Cognitive Theory**	Outcome expectancies (behavior)	12	0.75	4.30	0.76	8	0.70	3.82	0.89	9	0.80	3.56	0.67	7	0.56	4.96	0.73	6	0.76	6.01	1.19	3.56	4.30	6.01
	Outcome expectancies (self)	2	0.75	3.74	1.64									2	0.80	2.58	1.07					2.58	3.16	3.74
	Self efficacy	12	0.83	3.77	0.77	10	0.69	4.13	0.63	10	0.82	4.55	0.89	6	0.88	2.02	1.85	14	0.93	4.80	0.80	2.02	4.13	4.80
	Generalized self efficacy	10	0.87	3.00	0.37	10	0.83	2.99	0.37	10	0.87	3.05	0.38	10	0.85	2.86	0.36	10	0.87	2.80	0.40	2.80	2.99	3.05
**Implementation Intention**	Action Planning	1	-	5.40	1.60	1		5.10	1.50	1	-	5.15	1.59	1		5.10	1.70	1	-	5.60	1.60	5.10	5.15	5.60
**Learning Theory**	Anticipated consequences	2	0.51	4.65	1.30	3	0.51	4.30	1.00	3	0.42	4.84	0.89	3	0.61	5.07	0.93	2	0.46	5.80	1.00	4.30	4.84	5.80
	Evidence of habitual behavior	2	0.62	3.80	1.35	3	0.86	4.40	1.40	3	0.86	4.37	1.61	2	0.70	5.65	1.05	2	0.60	4.70	1.70	3.80	4.40	5.65
**Other**	Knowledge	5	0.20	4.40	0.80	7	0.01	2.70	1.30	7	0.00	3.30	1.10	5	0.00	2.90	0.90	5	0.21	3.1	1.00			

### Descriptive summary of variance explained

The proportion of variance in the three dependent variables of intention, behavioral simulation, and behavior explained by the theories for each of the five behaviors are shown in Table 4 and Additional file 4.

**Table 4:** Percentage of variance (R2) in intention, behavioral simulation, and behavior explained by theories

	**Dependent variable**	**Percentage of variance in DV explained by theory and constructs predicting behavior**													
		**TPB**			**SCT**			**II**			**LT**			**CSSRM**	**K**
**Taking Dental Radiographs (DRa)**	Intention	28***			39***			28***			43***			3	4**
	Behavioral simulation	16***			9***			10***			9***			3	0
	Behavior	13***			7**			11***			8***			0	0
		I, PBC(i), PBC(d)			SE, RP			AP			AC				
**Performing Dental Restorations (DRe)**	Intention	27.9***			21.4***			24.5***			24.5***			18.8	0
	Behavioral simulation	5.3**			13.1***			3.7			5.9*			0	0
	Behavior	1.1			0			0			0			0	5** K
**Placing Fissure Sealants (FS)**	Intention	30***			25***						58***			1	0
	Behavioral simulation	25***			28***			7**			30***			2	0
	Intention	30.2***			28.9***						42.6***			27.2***	2.3**
**Managing URTI (URTI)**	Behavioral simulation	26.7***			25.9***			6.2**			24***			16	4.5***
	Behavior	3.3*			4.9**			2.4			6.3*** H			2.8	0
		I, PBC(i), PBC(d)			RP, OE										
	Intention	25***			21.5***						26.3***			11.3***	2.3**
**Managing Back Pain (LBP)**	Behavioral simulation	11.6***			12.1***			1.5*			8.1***			3.6	0.5
	Behavior	0.4**			0.2			0			0.4			0	0
		Min	Med	Max	Min	Med	Max	Min	Med	Max	Min	Med	Max		
	Intention$	25	28	30.2	21.4	25	39				24.5	42.6	58		
		LBP	DRa	URTI	DRe	FS	DRa				DRe	URTI	FS		
	Behavioral simulation	5.3	16	26.7	9	13.1	28	1.5	6.2	10	5.9	9	30		
		DRE	DRa	URTI	DRa	DRe	FS	LBP	URTI	DRa	DRe	DRa	FS		
	Behavior$$	1.1	3.3	13	0	4.9	7	0	2.4	11	0	6.3	8		
		DRe	URTI	DRa	DRe	URTI	DRa	DRe	URTI	DRa	DRe	URTI	DRa		

*p < 0.05, **p < 0.01, ***p < 0.001.

*TPB* Theory of Planned Behavior, *SCT* Social Cognitive Theory, II Implementation Intentions, *LT* Learning Theory, *CSSRM* Common Sense Self Regulation Model, *K* Knowledge.

Constructs (with a statistically significant beta weight): *I* Intention, *PBC* Perceived Behavioral Control (I indirect or d direct), *SE* self efficacy, *RP* risk perception, *OE* outcome expectancies, *AP* action planning, *H* habit, *K* knowledge.

$ Prediction of intention by Implementation Intention not reported as II is a post-intentional construct.

$$ Back Pain not included as variance measured with McFadden’s R squared; Fissure Sealants not included due to problems measuring behavior.

Across all of the theories, the highest proportion of the variance explained was always for intention and the lowest was for objective behavior. The Knowledge-Attitudes-Behavior Model performed poorly across all five behaviors and three dependent variables. CSSRM performed poorly for predicting behavior, though it predicted intention for two of the behaviors. For TPB, SCT, II, and LT across the five behaviors, the theories predicted median R^2^ of 25% to 42.6% for intention, 6.2% to 16% for behavioral simulation, and 2.4% to 6.3% for (three of the five) objective measures of behavior.

Comparing the performance of the models, TPB was predictive of three behaviors (taking dental radiographs, managing URTIs without prescribing antibiotics, and managing low back pain), SCT and LT were predictive of (the same) two behaviors (taking dental radiographs and managing URTIs without prescribing antibiotics), action planning predicted one behavior (taking dental radiographs) while other models were not predictive of behavior.

The TPB, SCT, and LT performed well in predicting intention, confirming their effectiveness in predicting key elements of the models (behavioral intention in TPB; goals in SCT). However, LT predicted more variance in intention than either TPB or SCT for four of the five behaviors, despite having fewer predictive variables and not usually being used as a model of cognitions. The PAP ( Additional file 4) did not predict any dependent variable for ‘taking dental radiographs,’ predicted behavioral simulation and intention for ‘performing dental restorations,’ predicted intention for ‘placing fissure sealants,’ predicted all three dependent variables for ‘managing URTIs without prescribing antibiotics’ and predicted intention for ‘managing low back pain without ordering lumbar spine x-rays.’

## Discussion

We operationalized five theories plus II and Knowledge-Attitudes-Behavior model and conducted five large-scale postal questionnaire surveys across two professional groups of working healthcare professionals. We obtained responses covering the full range of the scales and, for three of the behaviors, we achieved the *a priori* sample size; our response rates were low for all five studies. The theories predicted varying amounts of the three study dependent variables, and there was variation in the amount of variance explained both across the dependent variables and across the five behaviors. Both volitional and non-volitional theoretical constructs predicted behavior.

### Strengths and weaknesses

A major strength of conducting five studies and having multiple theories/models operationalized in the same way across a wide range of behaviors that spanned disciplines was that this allowed the identification of variation in the performance of the models, both between models and across behaviors. Such variation would not be apparent from single condition, single theory studies. It allows an additional degree of interpretation that is not otherwise possible.

The fact that we used several theories/models and tried to adhere to the best practices for measuring constructs related to each theory (e.g., having three items measuring each construct to achieve a reliable measure of the construct) meant that the questionnaire was both long and appeared repetitive (e.g., When I see patients with URTIs, I automatically consider managing them without an antibiotic; it is my usual practice not to prescribe antibiotics for patients with URTIs; I am not to prescribe antibiotics for patients with URTI). In addition, the wording specified by each of the theories also meant that, for many questions the format of the questions may have appeared constrained and non-intuitive (e.g., the symptoms of an URTI are puzzling to me). This almost certainly affected our response rates.

We did not achieve our target number of responses for two of the surveys. This means that we may be underpowered for a number of our analyses and may underestimate the performance of the models. Higher response rates would give greater confidence in the representativeness of the data, though this aspiration is set against the evidence of modest and slowly declining response rates to postal surveys among healthcare professionals—61% over the 10 years up to 1995 and 57% in the 10 years after [26]. Increased response rates might be achieved by fully compensating the recipients for their time. While financial incentives have shown little impact in increasing survey response rates, the amounts involved have usually been small and not equivalent to full recompense [27]; it would be reasonable to explore the effectiveness of full recompense and to use it as a strategy in the future [28].

Although we used standard methods and wordings whenever possible, this was not possible for II and LT where constructs have more often been manipulated in experiments than measured in predictive studies such as those we have conducted. In addition, a number of the theories contained constructs that were similar and difficult to differentiate (e.g., TPB attitudes (indirect) and SCT outcome expectancies), and we ended up using the same questionnaire items as a measure of both.

### Specifying and measuring behavior

For any study of the behavior of healthcare professionals, there is the key decision about how to measure their behavior. We used measures of behavior that were available from routine sources. We were trading off any lack of specificity of these measures of behavior compared to the behaviors we aimed to predict against the costs of collecting the most specific data. This would have involved data collection within the practice of each recipient and would have been very costly.

We were aware that the concordance of the TACT specification of the behaviors in the questionnaires and the behavioral measures available within the various routine data sets varied. Of the five sets of behavior data, the three general dental measures were all claims-based measures from a comprehensive central claims database. We were confident that the dental radiographs data provided a good measure of the behavior we specified in the questionnaires, but there were problems with the other two dental behavior measures. The fee claim database was too imprecise to be used for the placing fissure sealants behavior (due to our interest in a specific age defined subgroup) and was not analysed, although a randomized controlled trial running during the time period of this study successfully collected practice based data on this behavior [29] and, using the same theories, successfully predicted behavior [30]. We were specifically interested in restorations in permanent teeth in children aged <17 years, but the dental restorations behavior data did not sufficiently specify this age group and tooth restored and so was less specific than we anticipated and would have wished.

### Performing or not performing target behaviors?

The two general medical behaviors we specified within the questionnaires were in the context of not performing a behavior—doctors managing URTI without prescribing antibiotics, and managing patients with low back pain without taking x-rays. Our objective measures of these behaviors were rates of prescribing antibiotics and performing x-rays. This meant that we could never know the number of patients who presented with URTI or back pain, or the proportion of those presenting who were not treated nor referred. We had to assume that this proportion varied across practices in the same way as the data we collected. It is also the case for these two behaviors that ‘not performing the target behavior’ is unlikely to reflect one or more common alternative behaviors across all clinician’s practice. Clinicians are likely to have a number of alternative behaviors that they perform in order to avoid prescribing or ordering x-rays rather than doing nothing. We have previously shown that generating alternative clinical behaviors can increase intention to ‘not perform’ behaviors that are counter to evidence-based practice [26]. Identifying and specifying these may have improved the precision of our prediction in these clinical areas—assuming we could also have identified a measure of them. Given the low proportions of variance in behavior explained (with the possible exception of dental radiographs), it would seem prudent to exercise caution about the use of routinely collected data unless it is a good match to the specified clinical behavior in the survey.

### Performance of the theories

The theories all predicted intention better than they predicted simulated behavior and, in turn, simulated behavior better than objective measures of behavior as is usually found [31] and is due at least in part to common method (self-report) variance. It seems logical that, as the length of the causal chain (attitudes to intention to self-report behavior to observable behavior) increases, there is more opportunity for other factors to come into play and prediction to be more imprecise.

The effectiveness of LT (and its consideration of habit) in predicting behavior raises the importance of considering non-volitional, non-deliberative aspects of performing clinical behaviors and the idea of dual processing models (i.e., models which propose both deliberative and associative or automatic processes) as explanatory models of professional behavior and as ways of considering analysing such data [26,27,32].

Prediction of behavior by knowledge was included because it is one of the most common implicit models as a basis for changing behavior used in medicine [29]; based on its poor performance, it would be easy to justify omitting it from further study and sensible to suggest that this is not a viable model for promoting the uptake of evidence-based practice. However, given that we only included five questions and may not have sampled the critical knowledge, there may be different measures of knowledge that are predictive. The CSSRM performed less well than other models. The CSSRM is model that was developed specifically in the context of an individual’s illness and ‘health’ behaviors and thus has a different standpoint to the other theories. We operationalized it to assess a clinician's view about a condition they themselves were not experiencing, but one that they had to treat. The poor results of this model over our studies suggest that clinicians’ perceptions about a condition, in and of itself, are unlikely to be taken into account when choosing the behavior to treat it.

### Implications

Several of the theories that we used performed well and should be further used in implementation research (TPB, SCT, II, LT). Given the length of any questionnaire using multiple models (and our experience of low response rates), it is important to consider ways of shortening the instruments. A number of simple steps seem worth considering. Researchers should consider removing models that do not consistently perform well (CSSRM and KAB) from these studies. In future, it might be possible to select theories that explain three key elements of behavior change: how motivation to change behavior is developed; how motivated individuals turn this motivation into action; and how behavior may be changed without the development of a deliberative intention to change.

It would also be important to include theories or models that suggest not only how to explain behavior change that has occurred, but also suggest how to change behavior; both LT and SCT give clear guidance on how to change behavior, while this is less clear for TPB. Further, II have been shown to be methods of changing behavior [32].

A further option as a prior step to developing an intervention could be to develop a screening instrument that uses single (or small numbers of) item measures of key constructs. For each behavior, an investigator could ask a single intention question, a single action question, and a single question tapping non-volitional processes in order to gain a profile of the influences on the behavior. The advantage of such a method is that the initial approach involves a very short instrument. If intention scores were high, there would be little additional benefit in measuring any pre-intentional measures because strengthening any of them could not produce a large increase in intention; under such circumstances, exploring further the post-intentional or contextual/organisational factors would be a better use of resources. A similar, though slightly longer approach would be to use the Theoretical Domains Framework [26,29,30,32,33] as an instrument to help identify potentially relevant domains and to target theoretical surveys addressing key domains.

Our studies represented a non-experimental empirical approach to understanding behavior and the processes involved in enacting them. They allowed the exploration of a range of theories and the potential for subsequent exclusion of theories that did not predict behaviors. They also allowed an understanding of both volitional and non-volitional processes. However, they do not have the ability to quantify behavior change or explain causal mechanisms, and to do that would require the use of experimental designs, either using interim outcomes [27,28,34] or actual clinical behaviors [35]. We have taken the ideas from these studies and one of these behaviors on to identifying and modelling the performance of an intervention [28,36]. Further, we have tested a clinical intervention based on LT, contrasting it with an intervention based on a knowledge model, in increasing dentists’ use of fissure sealants for children; the LT intervention, providing contingent consequences for each behavior, was effective in changing behavior, while the knowledge based intervention, providing additional education, did not influence behavior [35].

Within these five studies, and many other theory-based studies reported in the literature, the behaviors are those enacted by individuals in isolation from other healthcare professionals. However, much of the work of primary care medical practice involves the behaviors of teams of individuals (as in the management of chronic diseases) where patients are managed by the (more or less) integrated actions of teams of healthcare professionals, and this is where much future interest in the utility of theory-based methods will lie. With the unit of analysis at the level of the team (which in primary care is often the same as the practice), there is legitimate interest in the cognitions of all members of a team and the need to explore the impact of these on practice level behavior [37,38].

### Summary

We operationalized multiple theories measuring across five behaviors. Some of the theories that we used successfully predicted behavior offering a basis for choosing between them. Continuing challenges that emerge from our work are: the need for better specification of behaviors; to better operationalize theories; to consider how best to appropriately extend the range of theories; to further assess value of theories in different settings and groups; exploring the implications of these methods for the management of chronic diseases; and moving to experimental designs to allow an understanding of behavior change.

## Competing interests

MPE is Editor in Chief of Implementation Science; Jeremy Grimshaw, Liz Glidewell, and Nick Steen are Editorial Board members. All decisions on this paper were made by another editor.

## Authors’ contributions

MPE with GM, MJ and DB synthesized the data across the studies. MPE led the writing of the paper. All authors contributed on sequential drafts and approved the final draft.

## Supplementary Material

Additional file 1 Methods and Results for the behavior ‘Performing Dental Restorations’. Click here for file

Additional file 2 Questionnaire for the behavior ‘Performing Dental Restorations’. Click here for file

Additional file 3 Descriptive statistics for Common Sense Self Regulation Model for each of the five behaviors. Click here for file

Additional file 4 Percentage of variance in intention, behavioral simulation and behavior explained by theories and Performance of the PAP Model across the five behaviors. Click here for file
