# Supplementary material for: Explaining clinical behaviors using multiple theoretical models
Source: Implement Sci. 2012 Oct 17;7:99. doi: 10.1186/1748-5908-7-99 (PMC3500222; doi:10.1186/1748-5908-7-99)
Supplement: Additional file 1 — Methods and Results for the behavior ‘Performing Dental Restorations’. [file 1748-5908-7-99-S1.pdf]

Additional File 1.
Methods and Results for the behaviour “Performing Dental Restorations”

Methods

Design and participants

This was a predictive study. Theory based cognitions were assessed by postal questionnaire survey. Behavioural data was collected from itemised records of courses of treatment for NHS patients under 17 years of age of participating dentists over a 12 month period from the central fee claims database (MIDAS) at the Scottish Dental Practice Board. Planned analyses explored the predictive value of theory based cognitions in explaining variance in the behavioural data.

Study participants were a random sample of general dental practitioners (GDPs) from Scotland selected from the Scottish Dental Practice Board list by a statistician using a list of random sampling numbers.

Predictor measures

Theoretically derived measures were developed following standard operationalisation protocols wherever possible[1][2][3][4][5]. Questionnaire items were developed from initial interviews with 15 primary dental care practitioners in Scotland who took part in a semi-structured interview of up to 40 minutes. The interviews used standard elicitation methods and covered the views and experiences about restoring carious teeth in patients who were 17 years old or under. Responses were coded into belief domains (behavioural, normative, control) which were then used, in conjunction with the literature, to create the questions measuring theory constructs. Eight knowledge questions were developed by the study team based on restoration issues for which there was good evidence. Table 2 in the main paper provides a summary of the predictive measures used in this study; the instrument is available as Additional File 2. Unless otherwise stated, all questions were rated on a 7-point scale from *Strongly Disagree* to *Strongly Agree*. We aimed to include at least three questions per psychological construct.

Outcome measures

Behaviour

The behavioural measure was the total number of restorations per 100 courses of treatment, expressed as a percentage, calculated as the total number of restorations 6 months before and after the first posting of the questionnaire, divided by the total number of claims for that period.

Behavioural simulation

Key elements which might influence GDPs’ decisions to do a restoration were identified from the literature, opinion of the clinical members of the research team and the initial interviews with 15 GDPs. These elements were subdivided into three categories: Clinical (oral hygiene, previous restorations, clinically detectable caries, unrestored enamel lesions, extent of decay, success of previous work done in the mouth, pain); Clinician/Practice (Busy clinic, knowledge

---

[1] Ajzen I. The theory of planned behaviour. Organizational Behaviour and Human Decision Processes 1991; 50, 179-211.
[2] Conner M, Sparks P: The theory of planned behaviour and health behaviours. In Predicting health behaviour. Research and practice with social cognition models. 1st Ed edition. Edited by Conner M, Norman P. Buckingham: Open University Press; 1996:121-162.
[3] Francis JJ, Eccles M, Johnston M, Walker A, Grimshaw J, Foy R et al.: Constructing questionnaires based on the theory of planned behaviour: A manual for health services researchers. 2004.
[4] Bandura A: Self-efficacy: the exercise of control. New York: Freeman; 1997.
[5] Moss-Morris R, Weinman J, Petrie KJ, Horne R, Cameron LD, Buick D: The revised illness perception questionnaire (IPQ-R). Psychol Health 2002, 17: 1-16.

of patient, type of visit e.g. 6 monthly recall), Patient (age, anxiety, cooperative behaviour, attendance, attitude to teeth, attitude to dentist). From this five clinical scenarios were constructed describing patients presenting in primary care with dental caries (see Additional File 1). All should receive a restoration. Respondents were asked to decide whether or not they would do a restoration and decisions in favour were summed to create a total score out of a possible maximum of five.

Behavioural intention

Three questions assessed intention to restore: '*I aim to use restorations to manage caries in children under 17 years of age; I have in mind to use restorations when I see children under 17 years of age; I intend to restore teeth as a primary part of managing caries*. Responses were summed (range 3 – 21) and scaled so that higher scores reflected greater intention to restore.

Procedure

An independent statistician, using a list of random sampling numbers, selected 450 dentists from the Scottish Dental Board practice list. These dentists were sent an invitation pack (letter of invitation, questionnaire consisting of psychological and demographic measures and a consent form to allow access to their fee claims data from MIDAS, as well as a reply-paid envelope). Three postal reminders were sent to non-responders at 2 weeks, 4 weeks and 6 weeks from the first mailing (April to June 2004). Routinely collected data on fee claims for treatment, used to generate the primary outcome measure, were gathered for a 12 month period (6 months before and after the first questionnaire posting) to control for seasonal variations.

Sample size and statistical analysis

The target sample size was based on a recommendation by Green[6] to have a minimum of 162 subjects when undertaking multiple regression analysis with 14 predictor variables and an expected response rate of approximately 40% from previous surveys of this population.

The overall analytic approach was to first check the internal consistency of the measures. Where necessary questions were removed to achieve a Cronbach's alpha of 0.6 or greater. Where this was not possible the highest alpha was achieved. For two question constructs a correlation coefficient of 0.25 was used as a cut off. Next, for each of the three outcome measures, Pearson Correlation Coefficients between the individual constructs and the outcome measures were calculated and then multiple regression analyses were used to examine the predictive value of each theoretical model. For the five "perceived cause of illness" questions in the Common Sense Self-regulation Model responses were dichotomized into scores of five to seven (indicating agreement that the cause in question was responsible for caries) versus anything else (indicating disagreement). These dichotomous variables were then entered as independent variables into the regression. Finally, for predictors that were statistically significant, irrespective of whether or not they came from the same theory, we examined the relationship between predictive and outcome variables. All constructs which predicted the outcome were entered into a stepwise regression analysis to investigate the combined predictive value of significant constructs across all theories. The relationship between predictive and outcome variables were examined using ANOVA for the Stage Model. The relationship between Implementation Intention and intention was not explored as Implementation Intention is a post-intentional construct.

Ethics approval

The study was approved by the UK South East Multi-Centre Research Ethics Committee.

[6] Green SB: How many subjects does it take to do a regression analysis? Multivariate Behavioural Research 1991, 26: 499-510.

Results

Description of dataset

Response rate:	130/450 (29%) returned completed questionnaires. 116 had behaviour data.
Gender:	87 (67%) male.
Years qualified:	mean 19 (SD 8) years; median 19 (IQR 14 to 24) years.
List size:	Median 3000 (IQR 2000 to 6000).
Vocational Dental Training Scheme trainer:	17 (13%).
Median number of dentists in practice:	2 (IQR 1 to 3).
Median number of sessions worked per week:	8 (IQR 9 to 10).

Table 1 contains descriptive information on the behaviour data models. The descriptives are the means of the totals of the score of the items in the construct. As this is linear regression and we present beta, it doesn't make any difference (the information in a mean of means and a mean of totals is identical). A mean of means puts all the constructs on the same scale (apart from those that have a multiplicative component) making them easier to interpret.

In the tables, have used the * p< 0.05, ** p <0.01, *** p <0.001 throughout. r = Pearson correlation between the construct and the outcome variable (either behaviour, simulation or intention). Beta = the standardised regression coefficient, positive means a positive relationship, negative a negative relationship, the size of the coefficient measures the size of the relationship between the construct and outcome, adjusted for the other constructs in the model, as a shift in proportion of a standard deviation in the outcome for a shift of a standard deviation in the construct. The adjusted R^2 column gives the proportion of the variance explained by the model, the other columns after that are information in the regression (F statistic, and numerator and denominator degrees of freedom). The descriptive text in the table describes the constructs.

Theory of planned behaviour, none of the constructs were predictive of behaviour, no variance in behaviour was explained by this theory. In fact this was the case for all theories except the knowledge items The higher the knowledge score, the lower the behaviour, (there was a negative correlation between knowledge and behaviour), knowledge explained 5% of the variation in behaviour (this is from the adjusted R squared from linear regression).

Table 2 contains descriptive information on the behaviour simulation (BS) and intention data models. Intention was correlated with BS, as was the attitude indirect construct. The regression model with intention and PBC direct predicting BS predicted 5.3% of the variance in BS, adding in PBC power construct did not add to this model significantly (and PBC power was only weakly correlated with BS). Social cognitive theory explained 13.1% of the variation in BS, the outcome/expectancy and self efficacy constructs were the significant variables. Action planning explained 3.7% of the variation in BS. Evidence of habitual behaviour was the only predictive construct from operant learning theory; the theory explained 5.9% of the variance in BS. Self regulation model, and the knowledge construct did not predict BS. Stage theory explained 7.3% of the variance in BS.

Theory of planned behaviour predictor variables explained 27.9% of the variance in intention, the two attitude constructs were significant, the rest weakly correlated with intention. Social cognitive theory explained 21.4% of the variance in intention, as with BS, it was outcome/expectancy and self efficacy constructs were the significant variables. Implementation intention (action planning) explained 24.5% of the variation in intention. Operant learning theory explained 51% of the variation in intention, all the constructs were bivariately correlated with intention, but in the regression model only evidence of habitual behaviour and experienced consequences predicted intention (because anticipated consequences is correlated with both the other constructs in this theory.). Self regulation model explained 18.8% of the variance in intention. Stage theory explained 13.0% of the variance in intention. Knowledge items did not predict intention.

Table 3 shows the cross theory analysis. For behaviour the only significant predictor was knowledge so the results are the same as the individual theory section Table 1). Outcome/Expectancy and Self Efficacy together explained 14.2% of the variation in BS. Evidence of Habitual Behaviour, Attitude direct, Action Planning, Outcome expectancy, Anticipated consequences, Experienced consequences explained 61.6% of the variation in intention.

Table 1. Predicting behaviour by psychological theory: descriptive statistics, correlation and multiple regression analyses.

Theoretical framework	Predictive Constructs	N	Alpha	Mean	(SD)	r	Beta	R2(adj)	df	F
Theory of Planned Behaviour (a)	Attitude direct	2	0.37	9.4	(2.2)	-0.108				
	Attitude indirect	6	0.65	138.9	(33.9)	-0.023				
	Subjective Norm	4	0.77	61.2	(26.6)	0.024				
	Intention	3	0.79	14.7	(3.4)	-0.009	-0.001			
	PBC direct	10	0.71	14.3	(4.3)	-0.086	-0.108			
	PBC power	4	0.73	36.8	(8.1)	0.026	0.038	.011	3, 111	0.4
Social Cognitive Theory	Risk perception	3	0.51	12.9	(3.0)	-0.015	-0.036			
	Outcome expectancies	8	0.70	213.7	(50.0)	-0.005	-0.017			
	Self efficacy	10	0.69	41.3	(6.3)	0.100	0.113			
	Generalised self efficacy	10	0.83	29.9	(3.7)	-0.091	-0.088	0.00	4, 111	0.6
Implementation Intention	Action Planning			5.1	(1.5)	-0.025	-0.074	0.00	1, 114	0.1
Operant Learning Theory	*Anticipated consequences*	3	0.51	12.9	(3.0)	-0.015	-0.072			
	Evidence of habitual behaviour	3	0.86	13.2	(4.2)	0.114	0.148			
	Experienced consequences	4	-0.30	0.13	(0.77)	-0.013	-0.017	0.00	3, 112	0.7
Self-regulation Model	Identity of condition	2	0.15	6.7	(2.2)	0.124	0.123			
	Timeline acute	2	0.64	10.9	(2.5)	0.110	0.025			
	Timeline cyclical	2	0.50	7.5	(2.7)	0.022	-0.102			
	Control (treatment)	3	0.15	17.5	-(2.6)	0.088	-0.091			
	Control (patient)	3	0.64	11.4	(2.5)	0.020	0.052			
	Control (doctor)	2	0.17	10.6	(1.9)	-0.085	0.024			
	Cause a					0.023	0.075			
	Cause b					-0.104	-0.004			
	Cause c					0.021	0.114			
	Cause d					-0.163	0.069			
	Cause e					-0.052	-0.059			
	Consequence	3	0.46	15.7	(3.0)	-0.041	0.088			
	Emotional Response	4	0.65	14.4	(4.6)	0.009	-0.018			
	Coherence	2	0.69	4.8	(2.1)	-0.018	-0.135	0.00	14, 109	0.7
Stage Model	Behavioural stage[a]									
Other	Knowledge	7	.01	2.7	(1.3)	-0.250**	-0.250**	0.05	1, 114	7.6**

*p= or <0.05; ** p= or <0.01; ***p= or <0.001.

(a) Only intention and perceived behavioural control measures are entered into the regression equation as only these constructs are the proximal predictors of behaviour in this model. Alpha = Cronbach's Alpha; r = Pearson product moment correlation coefficient; Beta = standardised regression coefficients; - = single question measure. a) PAP stages were distributed as follows ; unmotivated 96(74%) , motivated more 1 (1%) , motivated less 4 (3%), action more 8 (6%), action less 19(15%) , not responded 2 (1%); ANOVA analysis showed that Behavioural stage did not predict the number of restorations performed: F(2,111) = 0.66, p = 0.521. Note: the stages have very small cells for an ANOVA; stage theory has 3 stages, unmotivated, motivated and action. These Stage theory results for behaviour are from ANOVA on percentages presented: behaviour F(4, 109) = 0.352, p = 0.842.

Table 2 . Predicting behavioural simulation and intention by psychological theory: correlation and multiple regression analyses.

		Behavioural simulation					Behavioural intention				
**Theoretical framework**	**Predictive Constructs**	**r**	**Beta**	**R2(adj)**	**df**	**F**	**r**	**Beta**	**R2(adj)**	**df**	**F**
	Attitude direct	0.124									
Theory of	Attitude indirect	0.225*									
Planned	Subjective Norm	-0.063									
Behaviour	Intention		0.259**								
	PBC direct		0.014	0.053	2, 126	4.6*					
	Intention	0.261**	0.250**								
	PBC direct	0.021	-0.036								
	PBC power	0.144	0.151	0.066	3, 125	4.0**					
	Attitude direct						0.534***	0.499***			
	Attitude indirect						0.303***	0.185*			
	Subjective Norm						-0.062	-0.070			
	PBC direct						0.160	0.046			
	PBC power						0.108	-0.082	0.279	5, 122	11.7***
	Risk perception	0.138	-0.053				0.289**	0.058			
Social Cognitive	Outcome expectancies	0.271**	0.236**				0.402***	0.315**			
Theory	Self efficacy	0.328***	0.292**				0.339***	0.255**			
	Generalised self efficacy	-0.070	-0.076	0.131	4, 125	5.8***	0.096	0.088	0.214	4, 124	9.7***
Implementation intention	Action Planning	0.211*	0.211*	0.037	1, 128	5.9*	0.495***	.495***	0.245	1, 127	41.2***
Operant Learning Theory	Anticipated consequences	0.138	0.016				0.289**	-0.062			
	Evidence of habitual behaviour	0.280**	0.263**				0.699***	0.674***			
	Experienced consequences	0.117	0.045	0.059	3,126	3.7*	0.344**	0.197*	0.510	3, 125	45.4***
	Identity of condition	0.092	0.123				-0.024	0.021			
	Timeline acute	0.068	0.025				0.167	0.008			
Self regulation	Timeline cyclical	-0.069	-0.102				-0.294**	-0.310**			
Model	Control (treatment)	-0.077	-0.091				-0.073	-0.073			
	Control (patient)	0.049	0.052				0.140	0.158			
	Control (doctor)	0.062	0.024				-0.080	-0.182			
	Cause a	0.063	0.075				-0.067	-0.026			
	Cause b	0.030	-0.004				0.139	0.163			
	Cause c	0.113	0.114				0.000	0.100			
	Cause d	-0.008	0.069				-0.179*	-0.124			
	Cause e	-0.072	-0.059				-0.134	-0.011			
	Consequence	0.113	0.088				0.125	0.231*			
	Emotional Response	-0.050	-0.018				-0.063	-0.013			
	Coherence	-0.145	-0.135	0.000	14, 109	0.7	-0.270**	-0.176	0.188	14, 108***	3.0
Stage Model	Behavioural stage[a]										
Other	Knowledge	-.050	-0.056	0.000	1, 128	0.4	0.016	0.016	.000	1,118	0.3

*p= or <0.05; ** p= or <0.01; ***p= or <0.001. r = Pearson product moment correlation coefficient; Beta = standardised regression coefficients. a) Stage theory ANOVA simulation F(4, 123) = 3.585, p 0.008, adjusted R^2 = 0.073; intention F(4, 122) = 5.637, p<0.001 adjusted R^2 = 0.130.

Table 3 Results of the stepwise regression analyses which included all constructs which significantly predicted outcomes.

**Outcome: Prescribing antibiotics**					
Predictive Constructs	***Entered***	***Beta***	***Adj. R^2***	***df***	***F***
Knowledge	Knowledge	-.0250**	0.05	1, 114	7.6**
**Outcome: Behavioural Simulation**					
TPB: Attitude Indirect; Intention<br>***SCT***: Outcome expectancy; Self Efficacy<br>***Implementation Intentions***: Action Planning<br>***Operant learning theory***: Evidence of Habitual Behaviour<br>***Stage Theory***	Outcome expectancy	0.297**			
	Self Efficacy	0.202*	0.142	2, 126	11.6***
**Outcome: Behavioural Intention**					
TPB: Attitude Indirect & Direct<br>***SCT***: Risk Perception; Outcome expectancy; Self Efficacy<br>***Implementation Intentions***: Action Planning<br>***Operant learning theory***: Anticipated Consequences; ; Evidence of Habitual Behaviour; Experienced Consequences<br>***Stage Theory***	Evidence of Habitual Behaviour	0.493***			
	Attitude direct	0.256***			
	Action Planning	0.169**			
	Outcome expectancy	0.200**			
	Anticipated consequences	-0.208*			
	Experienced consequences	0.140*	0.616	6, 121	34.9***

*p= or <0.05; ** p= or <0.01; ***p= or <0.001.
PBC = perceived behavioural control; TPB = Theory of Planned Behaviour; SCT = Social Cognitive Theory; SRM = Self-Regulation Model;
