# Supplementary material for: Explaining clinical behaviors using multiple theoretical models
Source: Implement Sci. 2012 Oct 17;7:99. doi: 10.1186/1748-5908-7-99 (PMC3500222; doi:10.1186/1748-5908-7-99)
Supplement: Additional file 2 — Questionnaire for the behavior ‘Performing Dental Restorations’. [file 1748-5908-7-99-S2.pdf]

MANAGING CARIES IN PATIENTS UNDER 17

First of all, thank you for participating in this research. This questionnaire contains items about elements that may influence your management of caries in **permanent** teeth in children, that is, patients under 17 years of age. The questionnaire items are based on a previous study of GDP attitudes about managing caries in clinical practice. You will notice that some of the questions are very alike – this is **not** meant to catch any one out. Sometimes questions posed in slightly different ways can mean different things to people, and this is something we are trying to understand - so please answer all the items without making a deliberate effort to be consistent. **This is not a test.** Dentists work in a wide range of contexts and treat patients from a variety of backgrounds, so we expect everyone to have different ideas and experiences, which will be reflected in their answers. The main thing to remember is to answer what first comes to mind, not what you believe you should answer or what we want to see – this is the only way progress can be made in understanding what is relevant in the 'real world'. Your responses will be held in confidence.

PARTICIPATION SLIP

Please mark the appropriate box

YES, I would like to participate in this study. I understand this means completing the questionnaire and giving permission for the PRIME research team to obtain data relating to restorations and practice profiles from MIDAS — 1. ☐

(please note that we need you to mark this box even if you have completed the questionnaire so that we can fully include your views in this study)

We appreciate that some participants would like to be informed about the end-result of projects that they are involved in. Please tick here if you would like to receive a summary of the results. ☐

- **NO**, I do not wish to participate in this study — 2. ☐

Signature…………………………………………… Date…..../…..../……...

Please check that your contact details are correct and amend if necessary.

Your Contact Phone Number ____________________ **e-mail** ____________________

This page will be removed and stored separately from the remainder of the questionnaire to preserve confidentiality

For office use only

BACKGROUND

Male ☐ Female ☐

1. How long have you been qualified as a dentist? ☐☐ **Years**

2 How many dentists (excluding you) are there in your practice? ☐☐

3. Are you now, or within the past 2 years been, a vocational dental trainer? **Yes** ☐ **No** ☐

4. How many clinical sessions (0.5 days) do you work per week on average? ☐☐

5. What is your ***approximate*** **total** practice list size? ☐☐☐☐☐☐

6. ***Approximately*** what proportion of your under 17 yr old patients do you consider to have a Moderate to High risk of caries? ____ % **Not sure** ☐

SECTION 1: THE USE OF RESTORATIONS IN MANAGING CARIES IN PERMANENT TEETH

Please treat all sub-questions, e.g. 1a) b) c), as separate questions and answer each one.

1. When managing caries in children under 17, rather than prevention, leaving, monitoring, or extracting the tooth:	*Strongly Disagree*						*Strongly Agree*
a) I feel under pressure from patients' parents to restore…………….	1	2	3	4	5	6	7
b) I feel under pressure from PG courses to restore…………………..	1	2	3	4	5	6	7
c) I feel under pressure from the Dental Practice Board to restore …	1	2	3	4	5	6	7
d) I feel under pressure from colleagues to restore………………..….	1	2	3	4	5	6	7
2. If I manage caries primarily through restorations, then I will think of myself as a caring dentist………………………………………..	1	2	3	4	5	6	7
3. If I manage caries primarily through restorations, then I will think of myself as a competent dentist…….…...……….……..…………	1	2	3	4	5	6	7
4. It is highly likely that children will be worse off if I do not manage their caries primarily by restoring..…...……….………….	1	2	3	4	5	6	7

5. In general, managing caries by restoring:	*Strongly Disagree*						*Strongly Agree*
a) Avoids problems with the restored tooth in the future…………..	1	2	3	4	5	6	7
b) Is stressful for the child……………………………………………..	1	2	3	4	5	6	7
c) Gives me a sense of achievement………………………………….	1	2	3	4	5	6	7
d) Has financial advantages over managing caries any other way…	1	2	3	4	5	6	7
e) May makes matters worse…………………………………………….	1	2	3	4	5	6	7
f) Is likely to influence the child's attitude *toward their teeth* for the better	1	2	3	4	5	6	7

g) Is likely to influence the child's attitude *toward their dentist* for the worse	**1**	**2**	**3**	**4**	**5**	**6**	**7**

6. From memory, ***approximately*** how many of the ***last ten*** of your child patients had a permanent tooth restored?

0	1	2	3	4	5	6	7	8	9	10

7. From memory, ***approximately*** what proportion of children *last month* had a permanent tooth restored? ________%

8a) For ***every ten*** children you see, how many would you ***expect*** to restore at least one permanent tooth?

0	1	2	3	4	5	6	7	8	9	10

b) ***Approximately*** what proportion of children you see *next month* would you ***expect*** restore at least one permanent tooth?.____________%

9. If I routinely restore a child's carious tooth:	***Strongly Disagree***						***Strongly Agree***
a) On balance, my life will be easier in the long run…………………	1	2	3	4	5	6	7
b) On balance, the consequences (e.g. stress, time, future treatment requirements) will make me generally worse off…………………..	1	2	3	4	5	6	7
10a) When I see a carious tooth, I automatically consider restoring rather than leaving or monitoring it…………………………………	1	2	3	4	5	6	7
b) When I see a carious tooth, I automatically consider restoring rather than using preventive measures……………………..……	1	2	3	4	5	6	7
11. It is my usual practice to restore a carious tooth in a child, rather than leaving or monitoring it………………………………………	1	2	3	4	5	6	7
12. I aim to use restorations to manage caries in children under 17 years of age ……………………………………………….……...	1	2	3	4	5	6	7
13. A preventive program aimed at controlling small lesions at the enamel stage would not be as successful as conventional restorations in managing caries of children ***in my practice***………	1	2	3	4	5	6	7
14. Currently, my standard method of managing caries teeth does not primarily include restoration………………………………………….	1	2	3	4	5	6	7

15a) Think about the last time you managed caries in a child by restoring a tooth and **felt pleased** that you had done so. In regard to managing caries, do you think the result of this episode has made you:

**More likely to restore**	☐	**Less likely to restore**	☐	**Unchanged**	☐	**Not sure**	☐	**Never occurred**	☐

b) Think about the last time you managed caries in a child by restoring a tooth and **felt sorry** that you had done so. In regard to managing caries, do you think the result of this episode has made you:

**More likely to restore**	☐	**Less likely to restore**	☐	**Unchanged**	☐	**Not sure**	☐	**Never occurred**	☐

c) Think about the last time you decided **not to** manage caries in a child by restoring a tooth and **felt pleased** that you had not done so. In regard to managing caries, do you think the result of this episode has made you:

**More likely to restore**	☐	**Less likely to restore**	☐	**Unchanged**	☐	**Not sure**	☐	**Never occurred**	☐

d) Think about the last time you decided **not to** manage caries in a child by restoring a tooth and **felt sorry** that you had not done so. In regard to managing caries, do you think the result of this episode has made you:

More likely to restore	☐	Less likely to restore	☐	Unchanged	☐	Not sure	☐	Never occurred	☐

16. In regard to managing caries in children, which of these sentences ***most*** characterises you at the moment

Please tick *only one* of the 7 boxes)

1.	i) I have not yet thought about changing the number of restorations that I currently do
2.	ii) It has been a while since I have thought about changing the number of restorations that I do
3.	iii) I have thought about it and decided that I *will not change* the number of restorations I do
4	iv) I have decided that I *will do more* restorations
5.	v) I have decided that I *will do less* restorations
6.	vi) I have *already* done something about increasing the number of restorations I do
7.	vii) I have *already* done something about decreasing the number of restorations I do

SECTION 2: MANAGING PATIENTS (UNDER 17 YRS)

1. I find it *difficult to decide* in favour of restoring if:	*Strongly Disagree*						*Strongly Agree*
a) The child is young (still has some deciduous teeth)………………	1	2	3	4	5	6	7
b) The patient is really frightened………………………………………	1	2	3	4	5	6	7
c) The parent wants me to do something different……………………	1	2	3	4	5	6	7
d) The parent is unmotivated when it comes their child's teeth…….	1	2	3	4	5	6	7
e) I think that doing something else would save trouble in the long run………………………………………………………………………	1	2	3	4	5	6	7
f) There is ***not*** a significant amount of decay in the tooth (i.e. not in the dentine)……………………………………………………………	1	2	3	4	5	6	7
g) There *is* a significant amount of decay in the mouth…………..…	1	2	3	4	5	6	7

2. I find it difficult to *effectively restore* a tooth in a child if:	*Strongly Disagree*						*Strongly Agree*
a) Their mouth is small………………………………………………..	1	2	3	4	5	6	7
b) Their oral hygiene is bad…………………………………………….	1	2	3	4	5	6	7
c) The child is distressed ……………………………..…………	1	2	3	4	5	6	7
3 a) I would like to manage caries in children by doing something other than restoring, but I don't really know if I can……………..	1	2	3	4	5	6	7
b) Whether I manage caries in children primarily by restoring is entirely up to me………………………………………………………	1	2	3	4	5	6	7
c) I am confident that I can restore carious teeth in children whenever I want ………………………………………………………	1	2	3	4	5	6	7
d) I can overcome all obstacles, whatever they may be, in regard to restoring carious teeth in children…………………………………..	1	2	3	4	5	6	7
e) I find it generally easy to restore carious teeth in children……..…	1	2	3	4	5	6	7
f) I find it generally difficult to manage caries in children except by restoring……………………………………………….…………..…	1	2	3	4	5	6	7
4 a) In general, the possible harm caused by restoring carious teeth in children is outweighed by its benefits………………………	1	2	3	4	5	6	7

b) In general, managing caries in children primarily by restoring is more often bad practice than good practice………………………	**1**	**2**	**3**	**4**	**5**	**6**	**7**
5. When a child has a carious tooth, I have in mind to restore……….	**1**	**2**	**3**	**4**	**5**	**6**	**7**
6. I intend to restore teeth as a primary part of managing caries……..	**1**	**2**	**3**	**4**	**5**	**6**	**7**

7. <u>In general</u>:

	Important						*Unimportant*
a) Avoiding problems with the restored tooth in the future is……..	1	2	3	4	5	6	7
b) Not causing the child stress is………………………………………	1	2	3	4	5	6	7
c) Having a sense of achievement is………………………………….	1	2	3	4	5	6	7
d) Not putting myself at a financial disadvantage is ……………..…	1	2	3	4	5	6	7
e) Not making matters worse by restoring is……………………….	1	2	3	4	5	6	7
f) Influencing a child's attitude *toward their teeth* for the better is	1	2	3	4	5	6	7
g) Not influencing a child's attitude *toward their dentist* for the worse is……………………………………………………………….	1	2	3	4	5	6	7
h) Thinking of myself as a caring dentist is………………………….	1	2	3	4	5	6	7
i) Thinking of myself as a competent dentist is…………………….	1	2	3	4	5	6	7

8. How confident are you that you can ***effectively*** restore a carious tooth in a child if:

	Not at all Confident						*Extremely Confident*
a) Their mouth is small…………………………………….……...	1	2	3	4	5	6	7
b) Their oral hygiene is bad…………………………………………….	1	2	3	4	5	6	7
c) The child is distressed …………………………………..………..	1	2	3	4	5	6	7

9. How confident are you that you can manage caries in children primarily by something ***different*** from restoring if:

	Not at all Confident						*Extremely Confident*
a) The child is young (still has some deciduous teeth)………………							
b) The patient is really frightened…………………………………….	1	2	3	4	5	6	7
c) The parent wants you to do something different…………………	1	2	3	4	5	6	7
d) The parent is unmotivated when it comes their child's teeth…….	1	2	3	4	5	6	7
e) You think that doing something else would save trouble in the long run……………………………………………………………….	1	2	3	4	5	6	7
f) There is ***not*** a significant amount of decay in the tooth (i.e. not in the dentine)……………………………………………………………	1	2	3	4	5	6	7
g) There *is* a significant amount of decay in the mouth…………..…	1	2	3	4	5	6	7

10. How motivated are you to do:

	Not at All						*Very Much*
a) What patients' parents think you should………………………….	1	2	3	4	5	6	7
b) What Post Graduate courses say you should……………………..	1	2	3	4	5	6	7
c) What the Dental Practice Board says you should…………………	1	2	3	4	5	6	7
d) What colleagues think you should…………………………………..	1	2	3	4	5	6	7

11a) Do you have a clear idea how you would *want* to manage caries in children? **Yes** ☐ **No** ☐ **Unsure** ☐

b) If ***Yes***, Please describe it briefly:

SECTION 3: CARIES

1. Caries is a condition:	*Strongly Disagree*						*Strongly Agree*
a) With symptoms generally of an intense nature……………….	1	2	3	4	5	6	7
b) With many symptoms …………………………………...…	1	2	3	4	5	6	7
c) Which should concern dentists……………………………….	1	2	3	4	5	6	7
d) Which is likely to be permanent rather than temporary……..	1	2	3	4	5	6	7
e) Which will pass quickly……………………………………….	1	2	3	4	5	6	7
f) Which is very unpredictable…………………………………..	1	2	3	4	5	6	7

2. Caries is caused by:	*Strongly Disagree*						*Strongly Agree*
a) Poor care from dentists in the past……………………………	1	2	3	4	5	6	7
b) Low exposure to fluoride ……………………………………….	1	2	3	4	5	6	7
c) Chance or bad luck…………………………………………….	1	2	3	4	5	6	7
d) Diet…………………………………………………………………	1	2	3	4	5	6	7
e) Poor oral hygiene…………………………………………….	1	2	3	4	5	6	7
3. The symptoms of caries change a great deal from day to day……..	1	2	3	4	5	6	7
4. There is very little that can be done to arrest the progress of caries.	1	2	3	4	5	6	7
5. There is nothing which can prevent caries…………………………...	1	2	3	4	5	6	7
6. Treatment can control caries…………………………………………..	1	2	3	4	5	6	7
7. There is a lot which the patient can do to control the symptoms or signs of caries………………………………………………………..	1	2	3	4	5	6	7
8. What the patient does can determine whether a) caries reverses	1	2	3	4	5	6	7
b) caries progresses	1	2	3	4	5	6	7
9. Nothing I do will affect the progression of caries…………………...	1	2	3	4	5	6	7
10. What I do can determine whether the patient's caries reverses…..	1	2	3	4	5	6	7
11. In general, caries is a serious condition……………………………..	1	2	3	4	5	6	7
12. Caries do not have much effect on a patient's life……………….	1	2	3	4	5	6	7
13. Caries can have serious financial consequences for the patient…..	1	2	3	4	5	6	7
14. I have a clear picture or understanding of caries…………………..	1	2	3	4	5	6	7
15. The symptoms of caries are puzzling to me………………………..	1	2	3	4	5	6	7
16. I get depressed when I think about patients suffering from caries	1	2	3	4	5	6	7
17. Seeing patients with caries does not worry me…………………….	1	2	3	4	5	6	7

18. Seeing patients with caries makes me feel angry…………………..	**1**	**2**	**3**	**4**	**5**	**6**	**7**
19. Seeing patients with caries can affect me emotionally (e.g. can make me feel helpless or distressed) ………………………………..	**1**	**2**	**3**	**4**	**5**	**6**	**7**

	True	False	Not sure
20 a) The most powerful single predictor of future caries increment in children is socio-economic group…………………………………………………………………………….	1	2	3
b) Restorations are recommended for routine use with high-risk children……….………	1	2	3
c) Restorations are appropriate when small dentine lesions are present………………….	1	2	3
d) The evidence suggests that one type of amalgam is better than another ……………..	1	2	3
e) Once applied, restorations only need attention if problems are encountered………..	1	2	3

21. *Approximately* how long does an average size restoration last in a child? ___________________________

22. *Approximately* how long would it take for a white spot carious lesion in the approximal surface of a molar to progress to the inner half of the dentine in the following circumstances:

a) For a 'typical' child under 17 years of age? *(Please circle your choice)*:

i) Less than 3 mths **ii) 3 to up to 6 mths** **iii) 6 mths to up to 1 yr** **iv) 1yr to 2 yrs** **v) More than 2 years**

b) For a child under 17 you classify as high risk? *(Please circle your choice)*:

i) Less than 3 mths **ii) 3 to up to 6 mths** **iii) 6 mths to up to 1 yr** **iv) 1yr to 2 yrs** **v) More than 2 years**

SECTION 4: DECISION-MAKING

The following scenarios include various elements that may influence your decision to manage caries in children by restoring. We ask you to address each scenario and make a final decision as to whether you would or would not do a restoration before the next regular maintenance visit. We have left space for you to comment on any aspect of a scenario, or your *decision, if you so choose.*

1. The first patient is a 10 year old girl who is new to the practice. On examination you see quite a bit of plaque and 2 unrestored dentine lesions. Her mother says she uses a fluoride toothpaste, but admits that the girl will try to get out of brushing if she can. When you raise the possibility of restoring, the mother gets quite upset, which visibly affects the anxiety of the child.

Restore? **Yes** ☐ **No** ☐

On the scale 1 to 10, how difficult was it for you to make a decision for this scenario?

Not at all	0	1	2	3	4	5	6	7	8	9	10	*Extremely Difficult*

If you wish to comment on this decision please do so here.

2. The next patient is a 15 year old boy, who you tend to see every 18 months or so. He came in on his own, as he has done for years. There is a borderline enamel/dentine lesion in an upper molar. Although he has a number of previous restorations, this visit you notice that there is a distinct change for the better in the general state of his mouth and his attitude to his teeth and diet. Given your knowledge of his family background, you feel as if its entirely up to you to encourage him to keep this up.

Restore? **Yes** ☐ **No** ☐

On the scale 1 to 10, how difficult was it for you to make a decision for this scenario?

Not at all	0	1	2	3	4	5	6	7	8	9	10	*Extremely Difficult*

If you wish to comment on this decision please do so here.

3. This patient is 8 years old, in for his regular recall, which you set last time at 6 months. Despite his regular attendance over the last 3 years and needing only one small filling in the past, he remains very fearful of treatment. He now has a lesion in an upper 6. He came into the clinic crying, and you find it impossible to tell if his distress is due to pain, or to seeing you. The general state of his mouth could definitely be better.

Restore? **Yes** ☐ **No** ☐

On the scale 1 to 10, how difficult was it for you to make a decision for this scenario?

Not at all	0	1	2	3	4	5	6	7	8	9	10	*Extremely Difficult*

If you wish to comment on this decision please do so here.

4. The next patient is a 12 year old boy, who you have been seeing regularly since he was 6. At his previous visit he had a lower 6 restored. You had also detected a small lesion in a newly erupted 7, which you decided to leave at that time. Although the lesion is larger now, he says he still is not experiencing any pain. His oral hygiene and his diet are excellent.

Restore? **Yes** ☐ **No** ☐

On the scale 1 to 10, how difficult was it for you to make a decision for this scenario?

Not at all	0	1	2	3	4	5	6	7	8	9	10	*Extremely Difficult*

If you wish to comment on this decision please do so here.

5. This patient is a 14 year old girl, who you have not seen before. Her oral hygiene is excellent, as is her diet. She is particularly proud of the fact that she has never had any previous work done. When you tell her she has a small lesion in one of her upper molars, she becomes very distressed and begs you not to do a restoration. You are not sure if you believe her when she says she isn't feeling any pain.

Restore? **Yes** ☐ **No** ☐

On the scale 1 to 10, how difficult was it for you to make a decision for this scenario?

Not at all	0	1	2	3	4	5	6	7	8	9	10	*Extremely Difficult*

If you wish to comment on this decision please do so here.

SECTION 5: YOUR GENERAL STYLE

**In general, how true are the following statements about you?**	*Not at all true*	*Barely true*	*Moderately true*	*Exactly true*
**1.** I can always manage to solve difficult problems if I try hard enough…	**1**	**2**	**3**	**4**
**2.** If someone opposes me, I can find means and ways to get what I want………………………………………………………………………..…	**1**	**2**	**3**	**4**
**3.** It is easy for me to stick to my aims and accomplish my goals………..	**1**	**2**	**3**	**4**
**4.** I am confident that I could deal efficiently with unexpected events.. ….	**1**	**2**	**3**	**4**
**5.** Thanks to my resourcefulness, I know how to handle unforeseen situations…………………………………………………………………..	**1**	**2**	**3**	**4**
**6.** I can solve most problems if I invest the necessary effort………………	**1**	**2**	**3**	**4**
**7.** I can remain calm when facing difficulties because I can rely on my coping abilities………………………………………………………………	**1**	**2**	**3**	**4**
**8.** When I am confronted with a problem, I can usually find several solutions……………………………………………………………………	**1**	**2**	**3**	**4**
**9.** If I am in a bind, I can usually think of something to do………………	**1**	**2**	**3**	**4**
**10.** No matter what comes my way, I'm usually able to handle it………..	**1**	**2**	**3**	**4**

Please return the completed questionnaire in the enclosed pre-paid envelope.
Thank you for your participation in this study, your contribution is very much appreciated..
