# Supplementary material for: Explaining clinical behaviors using multiple theoretical models
Source: Implement Sci. 2012 Oct 17;7:99. doi: 10.1186/1748-5908-7-99 (PMC3500222; doi:10.1186/1748-5908-7-99)
Supplement: Additional file 3 — Descriptive statistics for Common Sense Self Regulation Model for each of the five behaviors. [file 1748-5908-7-99-S3.pdf]

Additional File 3. Descriptive statistics for Common Sense Self-regulation Model for each of the five behaviours.

	Taking Dental Radiographs				Performing Dental Restorations				Placing Fissure Sealants					Managing URTIs					Managing Low Back Pain					
**Predictive Constructs**	**N**	**Alpha**	**Mean**	**SD**	**N**	**Alpha**	**Mean**	**SD**	**Constructs**	**N**	**Alpha**	**Mean**	**SD**	**Constructs**	**N**	**Alpha**	**Mean**	**SD**	**Constructs**	**N**	**Alpha**	**Mean**	**SD**	**% agree**
**Identity of condition**	3	0.21	0.67	0.13	2	0.15	3.35	1.10		2	0.38	3.64	1.26		2	1	3.70	1.00		3	0.49	4.20	0.80	
**Duration**																								
Timeline acute	4	0.44	4.68	1.00	2	0.64	5.45	1.25		2	0.46	5.50	1.12		1		3.60	1.20		2	0.19	3.40	0.80	
Timeline cyclical					2	0.50	3.75	1.35		2	0.42	3.49	1.35		1		3.70	1.30		3	0.54	4.40	0.90	
**Control by**																								
Overall	7	0.46	5.57	0.71																				
treatment					3	0.15	5.83	0.87		3	0.46	5.89	0.92		2	0	2.80	0.95		3	0.66	5.60	0.80	
patient					3	0.64	3.80	0.83		3	0.61	5.60	1.11		2	1	4.75	1.10		2	0.85	5.70	1.00	
Clinician					2	0.17	5.30	0.95		2	0.13	5.47	1.00		2	1	4.05	1.20		2	0.36	5.30	0.90	
**Cause**																								
Overall	5	0.28	4.32	0.68					Past care	1		2.67	1.49	Stress	1		3.70	1.40	Stress	1				126 (42)
									Exposure to fluoride	1		4.68	1.71	Chance/ bad luck	1		4.30	1.50	Chance/ bad luck	1				140 (47)
									Chance or bad luck	1		2.39	1.48	Social contact	1		5.20	1.00	Family problems	1				117 (39)
									Diet	1		6.59	0.82	viral prevalence	1		5.50	0.90	Poor prior med. care	1				66 (22)
									Oral hygiene	1		6.28	1.21	Air travel	1		4.70	1.20	Patient's behaviour	1				225 (85)
																			Ageing	1				217 (73)
																			Overwork	1				148 (49)
Consequence	4	0.53	3.88	0.78	3	0.46	5.23	1.00		2	0.41	4.93	1.22		2	0	4.00	1.50		2	0.21	4.80	0.80	
Emotional Response	4	0.70	3.23	1.20	4	0.65	3.60	1.15		4	0.65	3.58	1.11		4	1	2.40	0.93		4	0.69	5.10	1.00	
Coherence	4	0.64	1.08	0.45	2	0.69	2.40	1.05		2	0.52	5.76	1.01		2	1	5.55	0.90		2	0.74	2.70	1.00	
