# Supplementary material for: Explaining clinical behaviors using multiple theoretical models
Source: Implement Sci. 2012 Oct 17;7:99. doi: 10.1186/1748-5908-7-99 (PMC3500222; doi:10.1186/1748-5908-7-99)
Supplement: Additional file 4 — Percentage of variance in intention, behavioral simulation and behavior explained by theories and Performance of the PAP Model across the five behaviors. [file 1748-5908-7-99-S4.pdf]

Additional File 4.

Performance of the PAP Model across the five behaviours.

Taking Dental Radiographs	Groups at different stages did not differ in the number of radiographs actually taken (F(5, 162) = 0.76, p = 0.578), behavioural simulation score (F(6, 207) = 2.26, p<.05) or intention (F(6, 206) = 2.03, p = 0.063).
Performing Dental Restorations	The PAP model did not predict the number of restorations performed (F(2,111) = 0.66, p = 0.521); it did predict both behavioural simulation score (F(4, 123) = 3.585, p 0.008, adjusted R2 = 0.073) and intention (F(4, 122) = 5.637, p<0.001 adjusted R2 = 0.130).
Placing Fissure Sealants	The PAP model did not predict the decision to place a fissure sealant in the behavioural scenarios (F(3,116) = 0.90, p = 0.44) but did predict intention to place a fissure sealant (F(3, 119)= 5.66 p = 0.001).
Managing Upper Respiratory Tract Infections without prescribing antibiotics	GPs who endorsed that they had 'already changed my management of URTIs to try to avoid the use of antibiotics' issued a mean (95%CI) of 11.8 (21.1 to 2.5 , p = 0.014) fewer prescriptions than GPs who endorsed any other response. Similarly for scenario scores these GPs made a mean (95%CI) of1.0 (1.2 to –0.7, p <0.001) fewer decision to prescribe and their intention scores were significantly more positive (p <0.001).
Managing Low Back Pain without ordering Lumbar Spine X-rays	Comparing GPs who endorsed 'I have decided that I will request less lumbar X-rays I request' or 'I have already done something about decreasing the number of lumbar X-rays I request' the stage model did not predict behaviour or behavioural simulation but did predict intention (p<0.001).
